# Winter Temperature and Long-Term Mortality After Coronary Artery Bypass Grafting: A Multicenter Cohort Study

**DOI:** 10.3390/jcm15114216

**Published:** 2026-05-29

**Authors:** Tomasz Urbanowicz, Sleiman Sebastian Aboul-Hassan, Krzysztof Skotak, Maria Luszczyn, Łukasz Moskal, Jakub Bratkowski, Jarosław Bartkowski, Bartłomiej Perek, Mirosław Wilczyński, Krzysztof J. Filipiak, Romuald Cichoń, Marek Jemielity

**Affiliations:** 1 Department of Cardiac Surgery and Transplantology, Poznan University of Medical Sciences, 61-848 Poznan, Poland; 2 Department of Cardiac Surgery, MEDINET Heart Center Ltd., 67-100 Nowa Sol, Poland; 3 Department of Cardiac Surgery and Interventional Cardiology, Medical University of Zielona Gora, 65-046 Zielona Gora, Poland; 4 Institute of Environmental Protection, National Research Institute, 01-045 Warsaw, Poland; 5 Department of Cardiac Surgery, Central Clinical Hospital, Medical University of Lodz, 92-213 Lodz, Poland; 6 The Centre of Postgraduate Medical Education, 99/103 Marymoncka Street, 01-813 Warsaw, Poland

**Keywords:** CABG, OPCAB, temperature, winter temperature, environmental

## Abstract

**Background:** Traditional cardiovascular risk models focus on patient-related clinical variables, while the impact of long-term environmental exposure remains insufficiently characterized in post-revascularization populations. **Objective:** To evaluate the association between environmental exposure and long-term mortality after coronary artery bypass grafting (CABG), and to determine whether integrated environmental measures provide additional prognostic value beyond established clinical risk factors. **Methods:** This retrospective multicenter cohort study included 1042 consecutive patients undergoing CABG with a median follow-up of 8.1 years. Regional environmental data were linked to individual patients. Multivariable Cox regression models were constructed using a hierarchical approach. To address collinearity among environmental variables, principal component analysis (PCA) was applied. Mean winter temperature was analyzed as a clinically interpretable proxy of overall environmental exposure. **Results:** During follow-up, 220 deaths (21.1%) occurred. Established clinical predictors of mortality included age, diabetes mellitus, peripheral vascular disease, and dyslipidemia. Individual environmental variables showed unstable associations due to collinearity. PCA identified a dominant environmental component explaining 82.0% of variance; however, its association with mortality did not reach statistical significance after adjustment (HR 1.17 per SD, 95% CI 0.98–1.39; *p* = 0.083). In contrast, higher mean winter temperature was independently associated with increased mortality (HR 1.24 per SD, 95% CI 1.05–1.48; *p* = 0.013) per 1 °C increase, with evidence of non-linearity. **Conclusions:** Environmental exposure represents a relevant component of long-term risk after CABG. While individual environmental variables are highly correlated and unstable, clinically interpretable measures such as winter temperature may provide practical support for risk assessment.

## 1. Introduction

Long-term cardiovascular risk is traditionally conceptualized as a function of intrinsic patient characteristics, including comorbid burden, ventricular function, and procedural factors [1,2,3]. However, this paradigm assumes that, once coronary anatomy has been surgically treated, long-term outcomes are determined predominantly by patient-related clinical factors [4,5]. However, this view overlooks the possibility that cardiovascular outcomes are continuously modulated by external exposures that persist beyond the index intervention. This is particularly relevant in post-CABG patients, in whom substantial residual risk persists despite successful revascularization.

Environmental factors, including ambient temperature and air pollution, have emerged as important contributors to cardiovascular morbidity and mortality [6,7]. Epidemiological studies have demonstrated associations between environmental exposures and incident cardiovascular events, mediated by mechanisms such as endothelial dysfunction, oxidative stress, inflammation, and autonomic dysregulation [8,9,10,11]. Yet, these exposures have largely been studied as acute triggers or isolated variables, rather than as chronic, structured influences on disease trajectory [12,13].

This limitation reflects a broader conceptual gap. Environmental exposures are inherently correlated, cumulative, and multidimensional, which limits the ability of single-variable models to capture their overall impact [14,15,16]. As such, their effects are unlikely to be adequately captured by single-variable models. Instead, they may operate as latent exposure domains, reflecting underlying environmental structure rather than discrete causal agents.

Patients undergoing CABG provide a unique model for examining this hypothesis. In this population, focal coronary obstruction has been surgically corrected, and systemic processes, including vascular integrity, inflammatory burden, and metabolic regulation, shape long-term outcomes. Environmental exposure, acting continuously, may therefore assume a central role in determining long-term prognosis.

Environmental exposures are inherently correlated and are unlikely to act as independent variables; instead, they may reflect a structured exposure domain that influences cardiovascular risk. The aim of this study was to evaluate whether environmental exposure, considered as an integrated domain, is associated with long-term mortality after CABG, and whether it provides incremental prognostic information beyond established clinical risk factors.

## 2. Materials and Methods

### 2.1. Patients

This retrospective multicenter cohort study included 1042 consecutive patients undergoing CABG for multivessel coronary artery disease in Polish tertiary centers between 2005 and 2026. Only patients treated with both thoracic arteries revascularization were enrolled in the analysis.

To ensure a clinically homogeneous cohort, we applied predefined inclusion and exclusion criteria. Eligible patients were adults undergoing isolated coronary artery bypass grafting (CABG) for multivessel coronary artery disease.

Patients with concomitant valvular heart disease requiring surgical intervention were excluded to avoid confounding related to combined procedures. Similarly, patients with severely reduced left ventricular ejection fraction (LVEF < 30%) were excluded due to the dominant and independent impact of advanced ventricular dysfunction on long-term prognosis.

Additional exclusion criteria included active malignancy and chronic inflammatory or autoimmune diseases, as these conditions may independently influence survival, independent of cardiovascular risk.

Patients were followed for a median of 8.1 years (IQR 3.4–11.8). Only patients with complete clinical and environmental data were included in the final analysis.

Missing data were handled using complete-case analysis. The proportion of missing data for each variable was low (<1%) and considered unlikely to significantly affect the results. The number of patients included in each model is reported where applicable.

### 2.2. Outcomes

The primary endpoint was all-cause mortality (time-to-event). Cause-specific mortality data were not consistently available across centers; therefore, cardiovascular mortality was not analyzed separately. Moreover, cause-specific mortality data were not consistently available across participating centers; therefore, cardiovascular mortality could not be reliably assessed.

#### Modeling Strategy

A hierarchical modeling framework was applied:

1. Clinical baseline model
2. Individual environmental variables
3. Latent environmental exposure model

This approach allows separation of clinical risk from environmental signal and underlying exposure structure.

### 2.3. Environmental Exposure

Environmental variables were standardized (z-scores) and analyzed as continuous variables. Exposure data were assigned based on patients’ residential regions and averaged over the individual follow-up period. Non-linearity was assessed using categorical stratification and spline-based approaches. Environmental data were assigned based on patients’ residential regions and aggregated over the individual follow-up period, as presented in previous publications [7].

#### 2.3.1. Principal Component Analysis (PCA)

Principal component analysis (PCA) was applied to standardized environmental variables to derive orthogonal components representing integrated environmental exposure patterns. The first principal component (PC1), explaining the largest proportion of variance, was interpreted as a composite environmental exposure index.

#### 2.3.2. Secondary Analysis

Mean winter temperature was selected a priori as a clinically interpretable proxy of the latent environmental exposure axis. This variable was chosen not based on its loading magnitude in the PCA, but rather for its stability, physiological relevance, and representation of long-term thermal exposure.

### 2.4. Statistical Analysis

All statistical analyses were performed using R software (version 4.3.2; R Foundation for Statistical Computing, Vienna, Austria). Survival analyses were conducted using the “survival” and “survminer” packages. Principal component analysis (PCA) was performed using the “stats” package, and visualization and data handling were supported by “ggplot2” and “dplyr”.

Continuous variables are presented as median (interquartile range) or mean (standard deviation), as appropriate based on distribution assessed by visual inspection and Shapiro–Wilk testing. Categorical variables are presented as counts and percentages. Group comparisons were performed using the Mann–Whitney U test or Student’s *t*-test for continuous variables and the χ^2^ test or Fisher’s exact test for categorical variables.

Time-to-event analyses were performed using Cox proportional hazards regression models, with results expressed as hazard ratios (HR) and 95% confidence intervals (CI). The proportional hazards assumption was assessed using Schoenfeld residuals and graphical inspection. Continuous variables included in regression models were standardized (per 1 standard deviation) to facilitate comparability of effect sizes.

A hierarchical modeling strategy was prespecified to differentiate among clinical risk, environmental signals, and the latent exposure structure. Three sequential model classes were constructed:

1. Clinical baseline model, including demographic, comorbidity, and procedural variables
2. Expanded model with individual environmental variables, allowing assessment of direct associations
3. Latent environmental exposure model, derived using principal component analysis

This approach was specifically designed to address the high collinearity among environmental variables, which may lead to unstable estimates and misinterpretation when modeled simultaneously.

Principal component analysis (PCA) was applied to standardized environmental variables to derive orthogonal components representing latent exposure domains. Component selection was based on eigenvalues (>1), proportion of explained variance, and interpretability of loadings. The first principal component (PC1), which explained the majority of the variance, was selected as the primary latent environmental exposure variable and entered into multivariable Cox regression models.

Non-linearity of environmental exposures was assessed using restricted cubic spline functions and complementary categorical analyses (quartiles). Additionally, receiver operating characteristic (ROC) curve analysis was used to identify optimal thresholds based on the Youden index for Kaplan–Meier survival comparisons.

Model discrimination was evaluated using the area under the receiver operating characteristic curve (AUC). Sensitivity and specificity were calculated at the optimal cutoff point.

A prespecified secondary analysis evaluated mean winter temperature as a clinically interpretable proxy of the latent environmental axis. This variable was analyzed using continuous, categorical, and spline-based approaches to assess robustness and non-linearity of associations.

All tests were two-sided, and *p*-values < 0.05 were considered statistically significant.

## 3. Results

A total of 1042 patients undergoing CABG were included in the analysis, with a median follow-up of 8.1 years.

The survival group was defined as patients alive at the end of the follow-up period or censored at last contact, whereas the deceased group included all patients who experienced death from any cause during follow-up. Group classification was therefore based strictly on time-to-event outcomes within the survival analysis framework.

The survival and deceased groups encountered 822 and 220 participants, respectively, as shown in Table 1.

The parameters of environmental exposure were calculated individually for each patient based on the observation time. The detailed information is presented in the methods section. The comparison of environmental factors exposure for the survival and deceased groups is presented in Table 2.

In addition to the primary latent exposure analysis, we performed a prespecified secondary analysis focusing on mean winter temperature, selected a priori for its physiological relevance and relative stability compared with short-term temperature extremes.

### 3.1. Primary Findings

In multivariable Cox regression analysis, age, diabetes mellitus, peripheral vascular disease, and dyslipidemia were independently associated with increased long-term mortality. OPCAB surgery was associated with a reduced risk of mortality. These findings are consistent with established clinical risk profiles in post-CABG populations (Table 3).

Given the non-monotonic pattern observed across quartiles, additional analyses using restricted cubic splines were considered the primary method for assessing non-linearity.

#### 3.1.1. Environmental Variables in the Fully Adjusted Model

In the fully adjusted model including all environmental variables (Table 3), no individual environmental parameter demonstrated a consistent independent association with long-term mortality. Several environmental variables showed wide confidence intervals and unstable estimates.

These findings indicate substantial collinearity among environmental variables, limiting their interpretability when included simultaneously in multivariable models.

#### 3.1.2. Latent Environmental Exposure Structure

To address collinearity and identify underlying exposure structure, principal component analysis (PCA) was performed on 22 environmental variables. The first principal component (PC1) explained 82.0% of total variance, indicating a dominant latent environmental exposure axis (Table 4).

**Figure 1:**
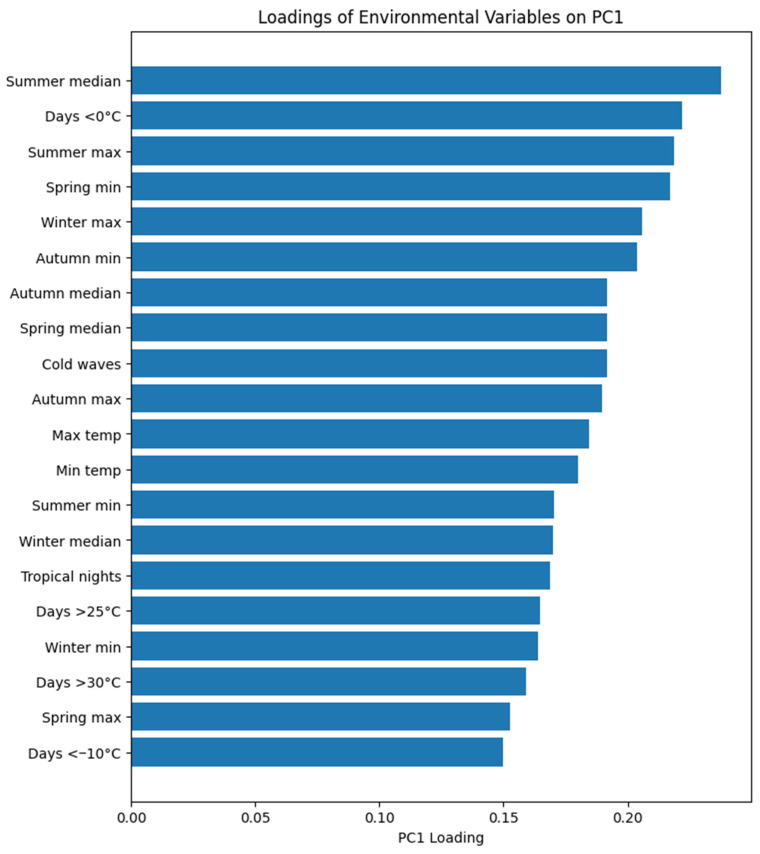
Loadings of environmental variables on the first principal component (PC1).

In multivariable Cox models adjusted for key clinical covariates, PC1 showed a positive but attenuated association with long-term mortality (HR 1.17 per SD, 95% CI 0.98–1.39; *p* = 0.083), suggesting a trend toward an association between integrated environmental exposure and long-term mortality. The loadings of environmental variables on the first principal component (PC1) were presented in Figure 1.

**Figure 2:**
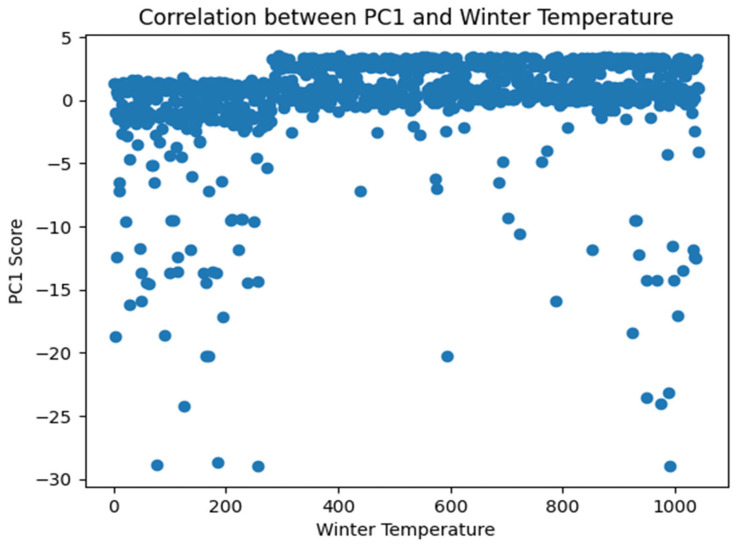
Correlation between PC1 and mean winter temperature.

In prespecified secondary analyses, mean winter temperature was evaluated as a clinically interpretable proxy of the latent environmental exposure domain. The mean winter temperature showed a modest positive correlation with PC1 (r = 0.27, *p* = 0.002), suggesting that it partially reflects the underlying environmental exposure axis (Figure 2).

#### 3.1.3. Model Discrimination

##### Model Performance

The observed model discrimination (AUC 0.658) indicates modest predictive performance, suggesting that environmental variables, while statistically associated with outcomes, provide limited incremental value beyond established clinical predictors. At the optimal threshold, sensitivity and specificity were approximately 62% and 63%, respectively.

##### Winter Temperature as a Translational Proxy

In prespecified secondary analyses, mean winter temperature was evaluated as a clinically interpretable proxy of the latent environmental exposure domain.

**Figure 3:**
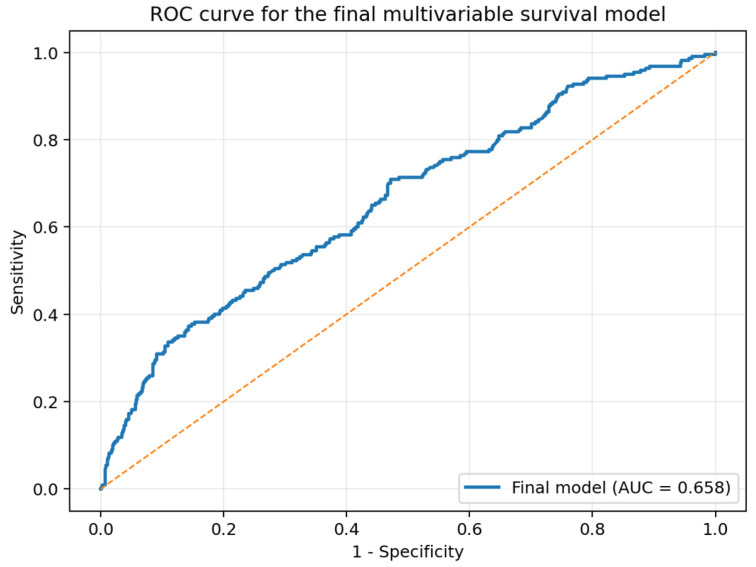
Receiver operating curve analysis for long-term mortality prediction.

Higher mean winter temperatures were independently associated with increased long-term mortality in multivariable Cox regression analysis (HR 1.24 per SD, 95% CI 1.05–1.48; *p* = 0.013) per 1 °C increase. This association remained consistent across multiple analytical approaches, including continuous modeling, categorical stratification, spline analysis, and ROC-derived thresholding (Figure 3).

**Figure 4:**
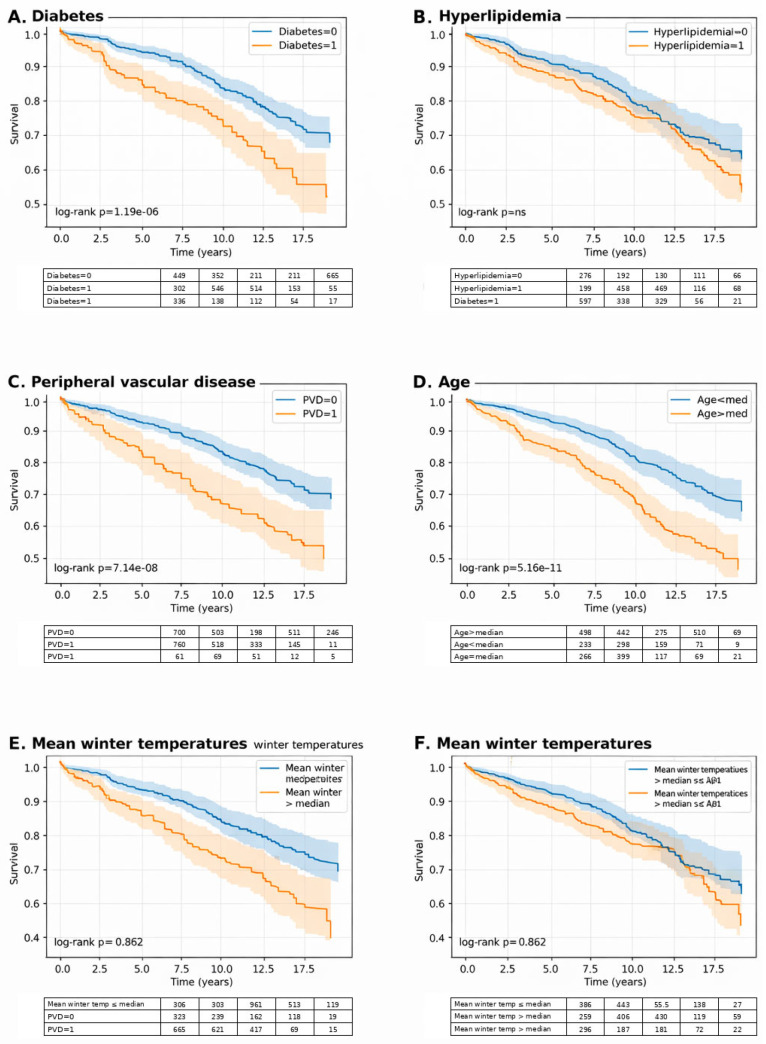
Kaplan–Meier survival curves for significant predictors of long-term mortality. Kaplan–Meier analysis for significant predictors of long-term mortality risk.

In multivariable Cox regression analysis, higher mean winter temperatures were associated with an increased risk of long-term mortality when modeled as a continuous variable. This association remained consistent after adjustment for key clinical covariates, including age, diabetes, hyperlipidemia, and peripheral vascular disease (Figure 4).

### 3.2. Secondary Findings

In secondary analyses, the mean winter temperature emerged as a representative and clinically interpretable environmental exposure.

In the fully adjusted Cox model, higher winter temperature was independently associated with increased long-term mortality:

- HR 1.24 per 1 °C increase (95% CI 1.05–1.48; *p* = 0.013)

Across multiple analytical approaches, including continuous modeling, categorical stratification, spline analysis, and ROC-derived thresholding, higher mean winter temperatures were consistently associated with increased long-term mortality. The relationship appears non-linear, with evidence of both threshold effects and variability across exposure levels.

#### Non-Linearity and Threshold Effects

Quartile-based analysis demonstrated a non-linear relationship between winter temperature and mortality. Compared with the lowest quartile, mortality risk was increased in the second (HR 1.49, 95% CI 1.03–2.17; *p* = 0.035) and fourth quartiles (HR 1.54, 95% CI 1.04–2.28; *p* = 0.031), but not in the third quartile.

Further stratification into quartiles revealed a non-uniform pattern:

- Q2 vs. Q1: HR 1.49 (95% CI 1.03–2.17; *p* = 0.035)
- Q3 vs. Q1: HR 1.18 (95% CI 0.79–1.77; *p* = 0.409)
- Q4 vs. Q1: HR 1.54 (95% CI 1.04–2.28; *p* = 0.031)

**Figure 5:**
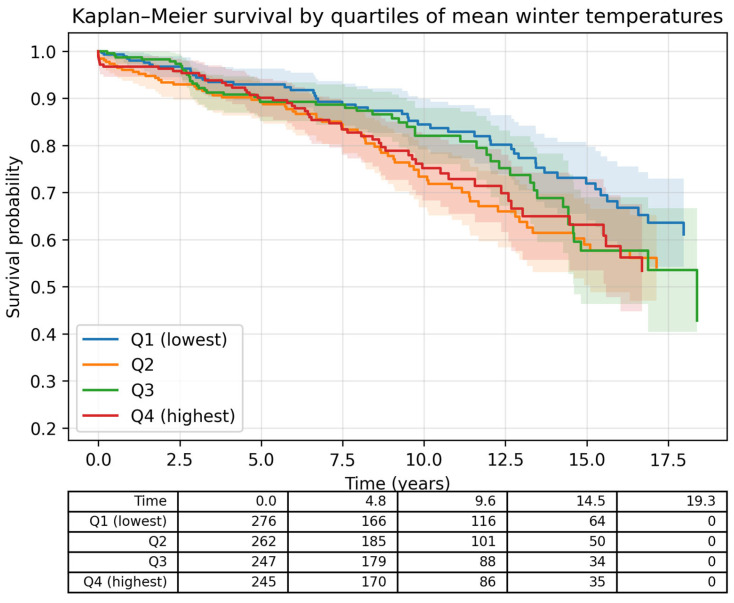
Kaplan–Meier survival in relation to mean winter temperature quartiles.

This pattern suggests a non-linear association, supported by the lack of a strictly monotonic increase across categories (Figure 5).

**Figure 6:**
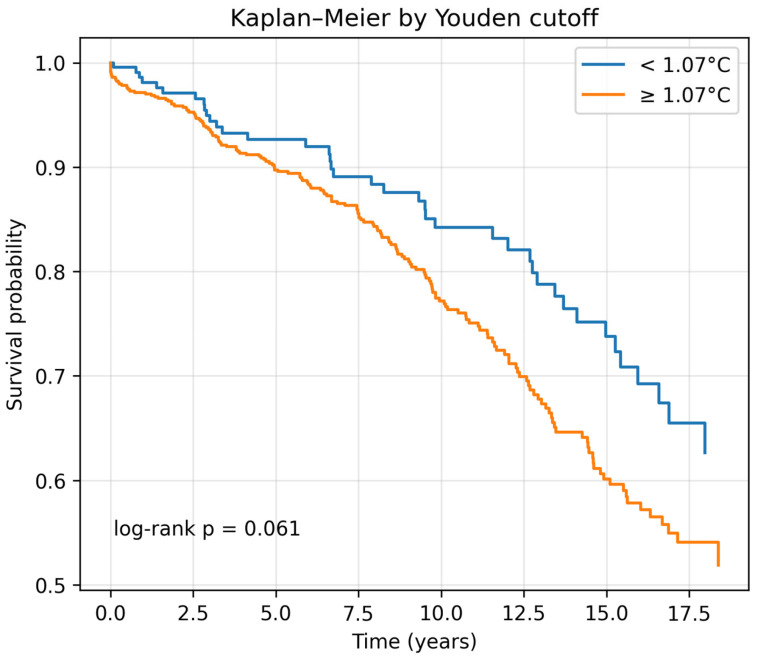
Kaplan–Meier analysis using the Youden cutoff for long-term mortality risk. *p* = 0.028.

Kaplan–Meier analysis using a Youden-derived cutoff (≥1.07 °C) demonstrated significantly reduced survival among patients exposed to higher mean winter temperatures, as presented in Figure 6.

## 4. Discussion

The principal finding of this study is that environmental exposure, when considered as an integrated construct, may contribute to long-term mortality risk after CABG. Importantly, these associations remained directionally consistent after adjustment for clinical factors, although the effect of the latent environmental component did not reach statistical significance. Chronic exposure to higher winter temperatures may influence cardiovascular risk through reduced cold-induced vasoconstriction, altered autonomic balance, and potential effects on physical activity patterns and metabolic regulation.

Importantly, winter temperature was not identified as a dominant variable within the PCA, but was selected as a clinically interpretable surrogate of the latent environmental exposure structure. This distinction reflects the difference between the statistical representation of exposure and its practical translation into clinically meaningful variables.

The apparent discrepancy between the non-significant association of the latent environmental component (PC1) and the significant association of mean winter temperature requires careful interpretation. PC1 represents a high-dimensional composite of correlated environmental variables, optimized for variance explanation rather than biological specificity. As such, it may dilute the effect of individual physiologically relevant exposures.

In contrast, mean winter temperature captures a specific and biologically meaningful aspect of environmental stress, particularly chronic thermal exposure, which has been linked to vascular tone regulation, autonomic balance, and inflammatory activation. Therefore, its association with mortality may reflect a targeted physiological pathway not fully captured by the multidimensional PCA construct, rather than a statistical artifact.

Our findings align with extensive epidemiological evidence linking environmental exposures to cardiovascular outcomes. This structured interpretation is consistent with observations from air pollution research, where individual pollutants such as NO_2_ and particulate matter exhibit strong interdependence and reflect shared emission environments rather than independent causal effects. Long-term exposure to NO_2_ and particulate matter has been associated with increased cardiovascular mortality in multiple large cohorts [17,18] and meta-analyses [19,20]. In previous reports, based on personalized air-pollution exposure after coronary artery bypass grafting, the possible impact of chronic exposure to nitric oxide and particulate matter has been postulated [21,22]. Similarly, temperature-related exposures have been shown to influence cardiovascular mortality, with both extreme heat and cold contributing to adverse outcomes [23,24,25]. The possible impact of low and warm ambient temperatures on premature, significant coronary culprit-lesion progression among patients with prior percutaneous interventions has been noted [26].

However, prior studies have largely focused on population-level risk, whereas our analysis extends these observations to a post-CABG cohort, a population characterized by advanced coronary disease and residual vulnerability. This distinction is critical, as environmental exposures in this setting may act not as initiators of disease, but as amplifiers of ongoing pathological processes.

A key conceptual contribution of this work is the framing of environmental exposure as a latent, multidimensional construct. The use of principal component analysis to derive a thermal exposure axis reflects the reality that environmental variables are highly correlated and rarely operate independently.

The observation that the mean winter temperature demonstrated a statistically significant association with mortality, whereas the latent environmental construct (PC1) did not, warrants careful interpretation. This apparent discrepancy reflects fundamental differences between statistical representation and biological specificity.

PC1 captures the dominant variance structure across highly correlated environmental variables and therefore represents a mathematically optimized but biologically diffuse construct. As such, it may dilute the effects of individual exposures that act through specific physiological pathways.

In contrast, mean winter temperature represents a targeted and physiologically meaningful exposure, closely linked to thermoregulation, vascular tone, autonomic balance, and seasonal behavioral adaptations. Its association with mortality may therefore reflect a specific mechanistic pathway not adequately captured by the multidimensional PCA framework.

Consequently, the predictive value of winter temperature should not be interpreted as contradictory to the latent model, but rather as a complementary, clinically interpretable proxy that captures biologically relevant aspects of environmental exposure. The observed model discrimination (AUC 0.658) indicates modest predictive ability. Importantly, this suggests that while environmental exposure demonstrates statistical associations with outcomes, its incremental clinical value beyond established risk factors remains limited. This finding highlights the distinction between etiological relevance and predictive utility. Environmental variables may contribute to long-term risk but are unlikely to substantially improve individual-level risk prediction when added to traditional clinical models.

Nonetheless, the possibility of a chance finding (Type I error) cannot be fully excluded, particularly given the multiple analytical approaches applied. However, the consistency of the association across continuous, categorical, spline-based, and threshold analyses argues against a purely spurious result and supports the robustness of the observed relationship.

This framework parallels observations in air pollution research, where NO_2_ and PM fractions often exhibit strong interdependence, reflecting shared emission sources such as traffic and combustion processes [27,28,29]. In such contexts, individual variables should not be interpreted in isolation, but rather as proxies capturing overlapping exposure domains.

A conceptual background linking environmental exposure to cardiovascular outcomes may involve several interrelated mechanisms. Chronic thermal exposure can influence vascular tone regulation, endothelial function, autonomic balance, and systemic inflammation. Additionally, environmental conditions may indirectly affect physical activity, dietary patterns, and exposure to pollutants, further modulating cardiovascular risk. These pathways likely interact with underlying patient vulnerability, amplifying risk in susceptible individuals rather than acting as isolated causal factors.

Potential mechanisms linking environmental exposure to cardiovascular outcomes include endothelial dysfunction, systemic inflammation, oxidative stress, and autonomic imbalance. These processes may contribute to progressive vascular disease and increased vulnerability in post-CABG patients [30,31,32,33,34,35,36,37,38,39]. These processes may contribute to progressive vascular disease and increased vulnerability in post-CABG patients.

The observed non-linear relationship between environmental exposure and mortality suggests that risk is not uniformly distributed across exposure levels. Instead, threshold-dependent effects may exist, beyond which physiological compensation becomes insufficient. The non-monotonic pattern observed across quartiles should be interpreted with caution. Such “zig-zag” relationships are unlikely to reflect true biological discontinuities and are more plausibly indicative of residual confounding or heterogeneity across exposure strata. Potential contributors include unmeasured regional differences in socioeconomic status, healthcare access, or comorbidity burden. Therefore, spline-based modeling, which provides a continuous and more physiologically plausible representation of exposure–risk relationships, should be considered the primary analytical approach for interpreting non-linearity.

This pattern is consistent with prior work demonstrating U-shaped or threshold relationships between temperature and mortality [40,41], and may reflect complex interactions among exposure intensity, duration, and individual susceptibility. The amplification of risk in patients with reduced ejection fraction highlights the role of physiological reserve as a modifier of environmental susceptibility. Patients with impaired cardiac function may be less able to compensate for environmental stressors, leading to disproportionate adverse effects. This supports a conceptual model in which environmental exposure interacts with underlying biological vulnerability, rather than acting independently.

### 4.1. Clinical Implications

These findings have several implications:

1. Environmental exposure should be considered in long-term risk assessment
2. Post-CABG management strategies may need to incorporate contextual environmental risk
3. Public health interventions targeting pollution and climate may have direct cardiovascular benefits in high-risk populations.

**Figure 7:**
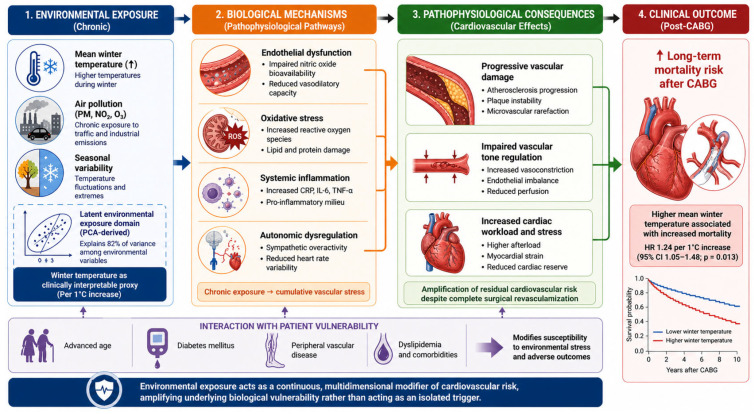
Interactions between environmental exposure, physiological pathways, and long-term cardiovascular risk, including the potential impact of winter temperatures after CABG.

A graphical summary of the interactions among environmental exposure, physiological pathways, and long-term cardiovascular risk may further facilitate the interpretation and clinical translation of these findings, as shown in Figure 7.

### 4.2. Limitations

This study has several limitations. First, the lack of systematic inclusion of echocardiographic parameters, particularly left ventricular ejection fraction, in the multivariable models (except for excluding patients with LVEF below 30%). Cardiac function is a well-established determinant of long-term outcomes after CABG and may interact with environmental exposure by modifying physiological reserve. Residual confounding related to variation in preserved or moderately reduced LVEF cannot be excluded and may influence susceptibility to environmental stressors. Second, environmental exposure was assigned at the regional level and may not reflect individual-level variability. Third, the observational design precludes causal inference. Fourth, collinearity among environmental variables limits the interpretation of individual effects. Fifth, the use of complete-case analysis may introduce selection bias. Finally, a key limitation of the present study is the use of all-cause mortality as the sole endpoint. Although this approach avoids misclassification bias, it limits mechanistic interpretation, as non-cardiovascular deaths may dilute associations specifically related to cardiovascular pathways. Future studies incorporating adjudicated cardiovascular mortality are warranted to better define the biological relevance of environmental exposure in this context.

## 5. Conclusions

Environmental exposure represents a clinically relevant component of long-term risk after CABG that is not captured by traditional clinical variables. Due to substantial collinearity, individual environmental factors should not be interpreted in isolation. Instead, integrated measures of environmental exposure may provide a more meaningful framework for risk assessment. Clinically interpretable proxies, such as mean winter temperature, may support the translation of environmental risk into clinical practice.

## Figures and Tables

**Table 1:** Groups characteristics.

Variables	Survival Group n = 822	Deceased Group n = 220	*p*
Demographics			
1. Age (years) (median (Q1–Q3))	58 (52–63)	61 (55–68)	<0.001
2. Sex (females) (n (%))	115 (13.9)	34 (15.5)	0.582
3. BMI (kg/m^2^) (median (Q1–Q3))	28 (26–31)	28 (25–30)	0.109
4. Obesity (BMI > 30) (n (%))	252 (30.7)	60 (27.3)	0.331
5. Active smokers (n (%))	231 (28.1)	52 (23.6)	0.183
Coronary disease history:			
1. Previous MI (n (%))	401 (48.8)	104 (47.3)	0.493
2. Previous PCI (n (%))	218 (26.5)	51 (23.2)	0.315
Co-morbidities			
1. Arterial hypertension (n (%))	401 (48.8)	102 (46.4)	0.524
2. Diabetes mellitus (n (%))	732 (89.1)	196 (89.0)	0.987
2.1. Insulin-dependent DM (n (%))	187 (22.8)	69 (31.4)	0.008
3. Dyslipidemia (n (%))	387 (47.1)	115 (52.3)	0.171
4. Renal impairment (n (%))	383 (46.6)	144 (65.6)	<0.001
4.1. Moderate * (n (%))	364 (44.3)	126 (57.3)	<0.001
4.2. Severe ** (n (%))	17 (2.1)	20 (9.1)	<0.001
4.3. Dialysis (n (%))	4 (0.5)	0 (0)	
5. Peripheral artery disease (n (%))	77 (9.4)	46 (20.9)	<0.001
6. Atrial fibrillation (n (%))	23 (2.8)	6 (2.7)	0.955
Procedural characteristics:			
1. Urgent/Emergency (n (%))	401 (48.8)	102 (46.4)	0.524
2. OPCAB surgery (n (%))	425 (51.7)	73 (33.2)	<0.001
3. Number of grafts (mean (SD))	3.00 (0.63)	3.00 (0.54)	0.363
4. Skeletonized ITA (n (%))	395 (48.1)	113 (51.4)	0.383
5. Hospital stay (days) (median (Q1–Q3))	7.0 (5.6–8.0)	7.0 (6.4–8.1)	<0.001

* Moderate renal impairment is defined by a glomerular filtration rate (GFR) below 60 but more than 30 mL/min/1.73 m^2^, ** Severe renal impairment defined by glomerular filtration rate (GFR) below 30 mL/min/1.73 m^2^, Abbreviations: BMI—body mass index, DM—diabetes mellitus, MI—myocardial infarction, n—number, OPCAB—off-pump coronary artery bypass grafting, PCI—percutaneous intervention, ITA—internal thoracic artery, Q—quartile.

**Table 2:** Environmental characteristics.

Variables	Survival Group n = 822	Deceased Group n = 220	*p*
Cold (median (Q1–Q3)):			
1. Cold waves * (days)	5.2 (5.0–5.3)	5.2 (5.1–5.3)	0.391
2. Days < 0 DC	21.1 (10.8–22.3)	21.1 (20.8–22.0)	0.157
3. Days < −10 DC	9.8 (9.2–10.4)	9.8 (9.2–10.3)	0.703
4. Minimal daily temperatures	5.6 (5.5–5.9)	5.6 (5.5–5.9)	0.206
Hot waves (median Q1–Q3)):			
1. Days > 25 DC	45.7 (43.8–46.9)	44.6 (44.2–47.2)	0.155
2. Days > 30 DC	11.4 (10.7–12.0)	11.5 (10.7–12.2)	0.087
3. Tropical nights ** (days)	3.6 (3.1–3.8)	3.6 (3.2–3.8)	0.179
4. Maximal temperatures	14.1 (13.9–14.2)	14.2 (14.0–14.2)	0.100
Seasonal temperatures (median (Q1–Q3)):			
Autumn (DC—Celsius degree)			
1. Maximal	14.5 (14.3–14.5)	14.5 (14.3–14.6)	0.058
2. Minimal	6.3 (6.2–6.6)	6.4 (6.2–6.7)	0.225
3. Median	10.4 (10.2–10.5)	10.4 (10.3–10.6)	0.118
Summer (Celsius degrees)			
1. Maximal	24.1 (23.9–24.2)	24.2 (24.0–24.2)	0.105
2. Minimal	6.3 (6.2–6.6)	6.4 (6.2–6.7)	0.225
3. Median	18.9 (18.7–19.0)	18.9 (18.7–19.0)	0.279
Spring (Celsius degrees)			
1. Maximal	14.1 (14.0–14.2)	14.2 (20.0–14.2)	0.192
2. Minimal	4.5 (4.3–4.7)	4.5 (4.4–4.7)	0.459
3. Median	9.1 (9.0–9.3)	9.1 (9.0–9.3)	0.382
Winter (Celsius degrees)			
1. Maximal	3.8 (3.5–3.9)	3.8 (3.5–3.9)	0.152
2. Minimal	−1.8 ((−)2.1–(−)1.5)	−1.7 ((−)2.0–(−) 1.5)	0.329
3. Median	1.3 (1.1–1.4)	1.2 (1.1–1.4)	0.317

* Cold waves—defined according to standard epidemiological criteria as periods of at least 3 consecutive days with a daily mean temperature below the 5th percentile of the local historical distribution. ** Tropical night—defined as a night when the minimum air temperature does not fall below 20 °C. Abbreviations: DC—Celsius degree, n—number.

**Table 3:** Multivariable model for long-term mortality risk in the analyzed population *.

Variable	HR (per SD or Category)	95% CI	*p*-Value
**Demographics**			
Age (per SD)	2.07	1.76–2.42	<0.001
Male sex	0.91	0.61–1.35	0.644
BMI (per SD)	0.93	0.72–1.19	0.544
Obesity (BMI ≥ 30)	0.97	0.59–1.58	0.888
Active smoking	1.18	0.84–1.67	0.331
Previous MI	1.22	0.91–1.64	0.190
**Comorbidities**			
Arterial hypertension	0.96	0.61–1.52	0.875
Diabetes mellitus	1.57	1.06–2.34	0.026
Insulin-dependent diabetes	1.56	0.94–2.58	0.087
Dyslipidemia	1.38	1.04–1.82	0.027
Renal impairment *	1.14	0.85–1.54	0.375
Peripheral vascular disease	1.87	1.31–2.66	<0.001
Atrial fibrillation	0.91	0.38–2.16	0.825
**Procedural variables**			
OPCAB vs. on-pump	0.68	0.49–0.95	0.022
Skeletonized ITA	1.18	0.83–1.66	0.355
**Environmental variables** **			
Cold waves (per SD)	0.91	0.71–1.16	0.443
Days < 0 °C (per SD)	0.77	0.18–3.20	0.716
Days < −10 °C (per SD)	1.81	0.76–4.32	0.184
Days > 30 °C (per SD)	1.10	0.32–3.78	0.875
Tropical nights (per SD)	1.25	0.82–1.89	0.295

* renal impairment is defined as glomerular filtration rate (GFR) below 60 mL/min/1.73 m^2^. ** Values are hazard ratios (HR) with 95% confidence intervals (CI) derived from a fully adjusted Cox proportional hazards model including all listed variables. Environmental variables were standardized (per 1 SD increase). Due to collinearity among environmental variables, effect estimates for individual environmental parameters should be interpreted with caution. Abbreviations: BMI—body mass index, °C—Celsius degree, CI—confidence interval, ITA—internal thoracic artery, HR—hazard ratio, MI—myocardial infarction, OPCAB—off-pump coronary artery bypass grafting, SD—standard deviation.

**Table 4:** PCA-derived latent environmental model.

Variable	HR	95% CI	*p*-Value
Age (per 1 SD)	1.97	1.71–2.28	<0.001
Peripheral vascular disease	1.94	1.39–2.70	<0.001
Diabetes mellitus	1.75	1.31–2.34	<0.001
Hyperlipidemia	1.46	1.11–1.92	0.007
PC1: latent environmental exposure axis (per 1 SD)	1.17	0.98–1.39	0.083

Abbreviations: CI—confidence interval, HR—hazard ratio, PC1—principal component 1, SD—standard deviation.

## Data Availability

Data supporting the reported results are available via e-mail upon reasonable request to the corresponding author.

## Abbreviations

The following abbreviations are used in this manuscript:

CABG	Coronary artery bypass grafting
OPCAB	Off-pump coronary artery bypass grafting
ITA	Internal thoracic artery
MI	Myocardial infarction
PCI	Percutaneous coronary intervention
BMI	Body mass index
DM	Diabetes mellitus
GFR	Glomerular filtration rate
HR	Hazard ratio
CI	Confidence interval
SD	Standard deviation
AUC	Area under the curve
ROC	Receiver operating characteristic
PCA	Principal component analysis
PC1	First principal component
DC	Degrees Celsius
